# Iron Storage within Dopamine Neurovesicles Revealed by Chemical Nano-Imaging

**DOI:** 10.1371/journal.pone.0000925

**Published:** 2007-09-26

**Authors:** Richard Ortega, Peter Cloetens, Guillaume Devès, Asunción Carmona, Sylvain Bohic

**Affiliations:** 1 Cellular Chemical Imaging and Speciation Group, Chimie Nucléaire Analytique Bioenvironnementale, Centre National de la Recherche Scientifique, Université Bordeaux 1, Gradignan, France; 2 X-Ray Imaging Group, European Synchrotron Radiation Facility, Grenoble, France; 3 Centro Nacional de Aceleradores, Universidad de Sevilla, Sevilla, Spain; 4 INSERM U-836, Equipe Rayonnement Synchrotron et Recherche Médicale, Grenoble Institut des Neurosciences, European Synchrotron Radiation Facility, Grenoble, France; Swiss Federal Institute of Technology Lausanne, Switzerland

## Abstract

Altered homeostasis of metal ions is suspected to play a critical role in neurodegeneration. However, the lack of analytical technique with sufficient spatial resolution prevents the investigation of metals distribution in neurons. An original experimental setup was developed to perform chemical element imaging with a 90 nm spatial resolution using synchrotron-based X-ray fluorescence. This unique spatial resolution, combined to a high brightness, enables chemical element imaging in subcellular compartments. We investigated the distribution of iron in dopamine producing neurons because iron-dopamine compounds are suspected to be formed but have yet never been observed in cells. The study shows that iron accumulates into dopamine neurovesicles. In addition, the inhibition of dopamine synthesis results in a decreased vesicular storage of iron. These results indicate a new physiological role for dopamine in iron buffering within normal dopamine producing cells. This system could be at fault in Parkinson's disease which is characterized by an increased level of iron in the substancia nigra pars compacta and an impaired storage of dopamine due to the disruption of vesicular trafficking. The re-distribution of highly reactive dopamine-iron complexes outside neurovesicles would result in an enhanced death of dopaminergic neurons.

## Introduction

The complete understanding of what is the chemical basis of the neuron cells is a major challenge that requires the development of new single-cell analytical methods. For example, very little is known about the distribution of metal ions such as iron, zinc, or copper, in neurons at the subcelullar level. However, those chemical elements have essential regulatory functions and their disturbed homeostasis is involved in neurodegenerative diseases [1], [2] such as Alzheimer's disease, Huntington's disease, or Parkinson's disease (PD). Anomalous iron handling has been proposed to be involved in the selective loss of dopaminergic neurons from the substancia nigra pars compact (SNpc) in PD [3], [4]. Indeed, iron specific accumulation in the SNpc is associated with PD [5]–[7]. This phenomenon is still unexplained. The role of iron in the etiology of PD is also supported by pharmacological evidence; a number of iron chelators have been shown to attenuate PD symptoms in animal models [2], [8], [9], confirming that iron could either mediate or accentuate neurotoxicity. Because dopamine can form stable complexes with iron *in vitro* [10], [11], it has been suggested that dopamine may exert a protective effect by chelating iron in dopaminergic neurons and that this system might be at fault in PD [12]. It is therefore theoretically possible that dopamine-iron complexes may exist in dopaminergic neurons but they have not yet been evidenced experimentally. The question then arises whether the dopamine-iron complex occurs *in vivo* and if so, where?

**Figure 1:**
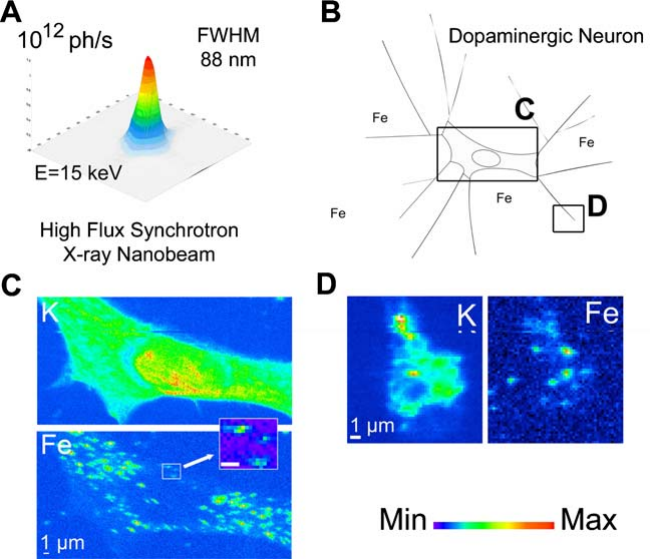
Synchrotron X-ray chemical nano-imaging reveals iron sub-cellular distribution. The synchrotron X-ray fluorescence nanoprobe end-station installed at ESRF was designed to provide a high flux hard X-ray beam of less than 90 nm size (FWHM, full width at half maximum). The intensity distribution in the focal plane is shown in (A); dopamine producing cells were exposed *in vitro* to 300 µM FeSO_4_ during 24 h (B). Chemical element distributions, here potassium and iron, were recorded on distinct cellular areas such as cell bodies (C), neurite outgrowths, and distal ends (D). Iron was found in 200 nm structures in the cytosol, neurite outgrowths, and distal ends, but not in the nucleus. Iron rich structures are not always resolved by the beam and clusters of larger dimension are also observed. Min-max range bar units are arbitrary. Scale bars = 1 µm.

Chemical imaging is the simultaneous measurement of chemical information and spatial information. Up to now the lack of analytical techniques with sufficient spatial resolution and detection sensitivity prevented the study of iron distribution in neurons at the subcelullar level. We developed an original setup for high spatial resolution chemical imaging at the European Synchrotron Radiation Facility (Fig. 1A, B) with a 88 nm X-ray beam of very high flux (up to 10^12^ photons/s). This spatial resolution is ten times better than what was available up to now for hard X-ray chemical imaging [13]. The characteristics of this unique nanoprobe fulfill the requirements for mapping biological trace element distributions at a size compatible with the analysis of most cellular compartments such as mitochondria, lysosomes, or neurosecretory vesicles. As exemplified in Figure 1, this newly developed synchrotron X-ray fluorescence nanoprobe is able to detect down to 10^−18^ g of Fe within a cellular structure as small as 100 nm diameter. The aim of this study was to elucidate the role of dopamine on iron homeostasis in dopaminergic cells by comparing dopamine and iron distributions in control dopaminergic cells and dopaminergic cells exposed to an inhibitor of dopamine synthesis. Using this chemical nano-imaging system we investigated the subcellular distribution of iron in dopamine producing neurons. PC12 rat pheocromocytoma cell line was used as *in vitro* model of dopamine producing cells [14], [15]. PC12 neuronal cells were differentiated with NGF and exposed *in vitro* to sub-cytotoxic concentrations of iron and/or alpha-methyltyrosine (AMT), an inhibitor of tyrosine hydroxylase (TH), the rate limiting enzyme in the biosynthesis of dopamine.

## Results

### Iron and dopamine distribution in dopamine producing cells

**Figure 2:**
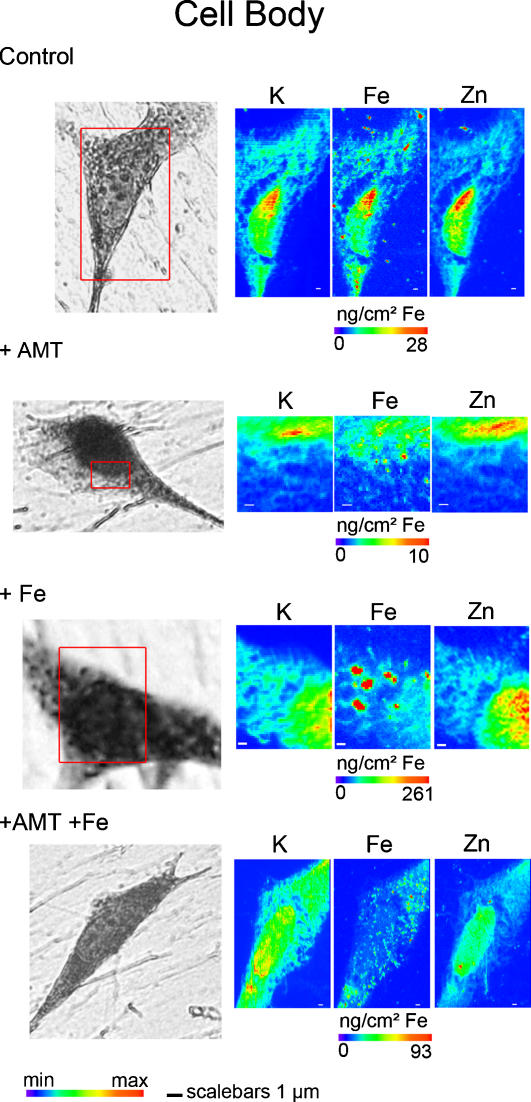
Nano-imaging of potassium, iron, and zinc in cell bodies. Each series of images are representative of the entire cell population for each condition (control, 1mM AMT and/or 300 µM FeSO_4_). The scanned area (left images, red squares) is shown on a bright field microscopy view of the freeze dried cell. Iron is located within the cytosol in vesicles of 200 nm size or more (Control). In cells exposed to iron alone Fe, and to AMT+Fe, a larger number of iron-rich structures are observed in cell bodies. In cell bodies of cells exposed to AMT alone, only a basal level of diffused iron is observed and almost no iron-rich structures. Min-max range bar units are arbitrary for potassium and zinc distributions. For iron distribution the maximum threshold values in micrograms per squared centimeter are shown for each color scale. Scale bars = 1 µm.

**Figure 3 pone-0000925-g003:**
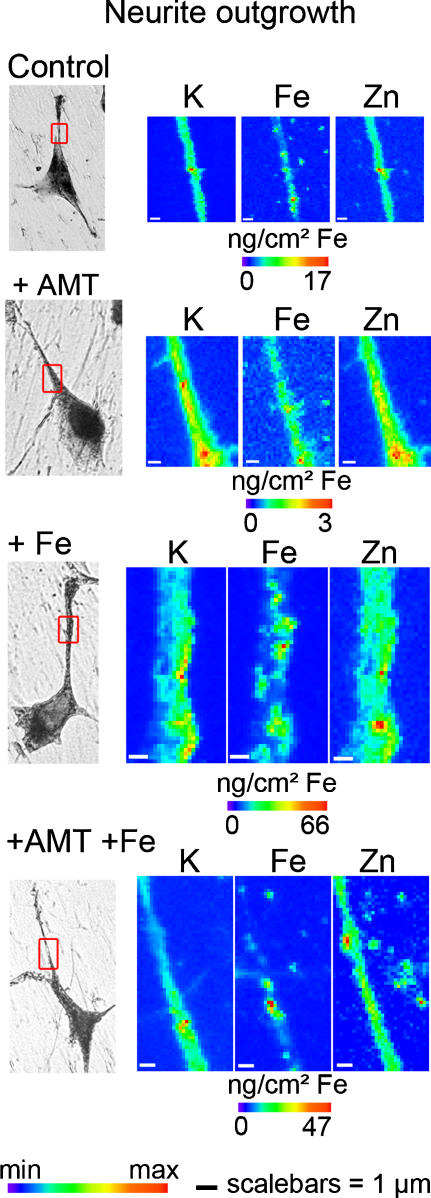
Nano-imaging of potassium, iron, and zinc in neurite outgrowths. Each series of images are representative of the entire cell population for each condition (control, 1mM AMT and/or 300 µM FeSO_4_). The scanned area (left images, red squares) is shown on a bright field microscopy view of the freeze dried cell. Iron is located within dopamine vesicles of 200 nm size or more in control cells with a large number of Fe-dopamine structures in Fe exposed cells. Iron concentration is close to the limit of detection in neurites of AMT cells. Min-max range bar units are arbitrary for potassium and zinc distributions. For iron distribution the maximum threshold values in micrograms per squared centimeter are shown for each color scale. Scale bars = 1 µm.

**Figure 4:**
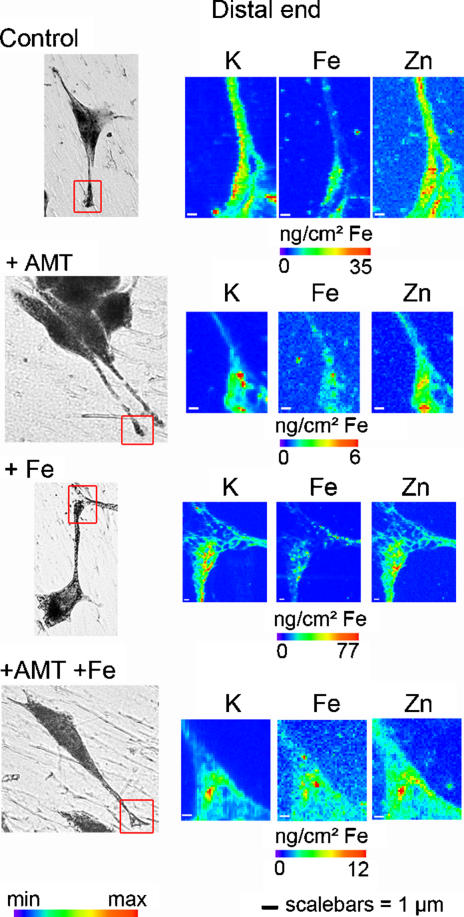
Nano-imaging of potassium, iron, and zinc in distal ends. Each series of images are representative of the entire cell population for each condition (control, 1mM AMT and/or 300 µM FeSO_4_). The scanned area (left images, red squares) is shown on a bright field microscopy view of the freeze dried cell. Iron is located within dopamine vesicles of 200 nm size or more (Control, and Fe conditions). Iron concentration is close to the limit of detection in distal ends of AMT, and AMT+Fe cells; only a basal level of Fe is observed. Min-max range bar units are arbitrary for potassium and zinc distributions. For iron distribution the maximum threshold values in micrograms per squared centimeter are shown for each color scale. Scale bars = 1 µm.

**Figure 5:**
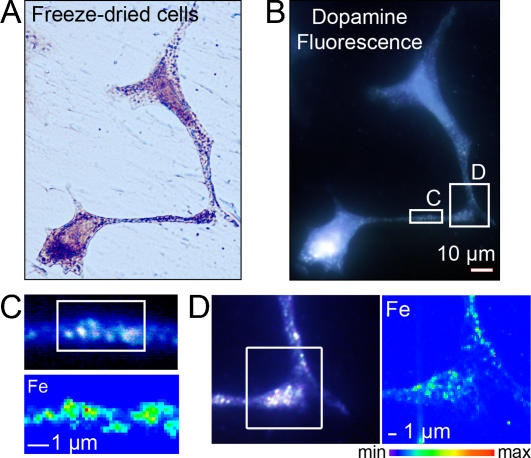
Iron is localized within dopamine neurovesicles. Visible light microscopy of freeze-dried cells (A), and epifluorescence microscopy of the same freeze-dried cells (B) enable the identification of dopamine distribution, while synchrotron X-ray fluorescence nano-imaging reveals the distribution of iron (C, D). Panels C and D represent comparison of the same region imaged in a fluorescent mode to visualize dopamine and with X-ray fluorescence to localize iron. Dopamine and iron are co-located within 200 nm structures characteristic of dopamine neurovesicles as identified by epifluorescence microscopy. A large number of iron and dopamine neurovesicles are found in neurite outgrowths (C) and distal ends (D). Min-max range bar units are arbitrary. Scale bars = 1 µm.

The iron profile distribution, as retrieved for example from the region zoomed in figure 1C, shows that iron is localized nearly exclusively in structures of typically 200 nm in size. Iron rich structures are not always resolved by the beam and clusters of larger dimension are also observed. These structures are found in the cytosol (Fig. 2), neurite outgrowths (Fig. 3), and distal ends (Fig. 4) of dopamine producing PC12 cells. The combination of dopamine fluorescence microscopy and synchrotron X-ray chemical nano-imaging reveals the co-localization of iron and dopamine in dopamine neurovesicles (Fig. 5C, D). A blue fluorescence was observed, only for cells exposed to an excess of iron, which corresponds to the reported fluorescence of oxidized forms of dopamine [16], [17]. When control cells are compared to cells exposed to an excess of iron, the same subcellular distribution is found but with a higher number of iron-rich structures in iron-exposed cells (Fig. 2). The iron content is found particularly high in neurite outgrowths and distal ends of cells exposed to excess iron (Fig. 3 and 4). Using the multielemental capabilities of the synchrotron X-ray fluorescence nanoprobe we were also able to image the distribution of some other essential chemical elements such as potassium or zinc (Fig. 2 to [Fig pone-0000925-g003] 4). Contrary to what is observed for iron, potassium and zinc are ubiquitous in dopamine producing cells and not selectively distributed to dopamine neurovesicles.

### Inhibition of dopamine synthesis

**Figure 6:**
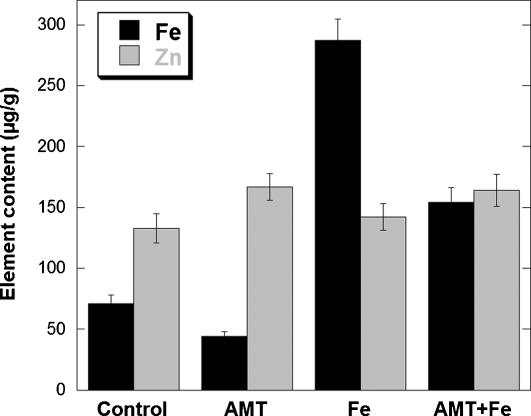
Cellular iron and zinc concentrations (µg/g dry mass; mean±SD; n = 6), obtained by PIXE quantitative micro-analysis. The data are the mean of six independent analyses performed on areas containing several hundred of cells for each condition of culture. The inhibition of dopamine synthesis (AMT, and AMT+Fe) results in a decrease of total iron concentration, while zinc concentration is not changed, suggesting a specific role of dopamine in iron homeostasis.

**Figure 7:**
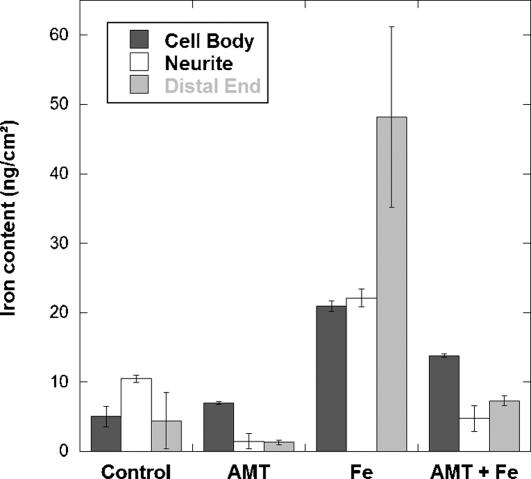
Sub-cellular iron quantitative distribution in cell bodies, neurite outgrowths, and distal ends (ng/cm^2^ ; mean±SD; n = 4 to 6), obtained through synchrotron X-ray fluorescence nanoprobe analysis shows that iron content is decreased particularly in neurite outgrowths and distal ends after AMT exposure (AMT and AMT+Fe), indicating that the decrease of total iron concentration is related to the reduction of the number of iron-dopamine neurovesicles.

To confirm the interaction of dopamine and iron, PC12 cells were exposed to AMT an inhibitor of TH and consequently of dopamine synthesis. The inhibition of TH results in a decrease of the total iron content in PC12 cells (Fig. 6). The decrease of iron consecutive to AMT treatment was observed both in absence or presence of excess iron. It is interesting to note that AMT had no effect on the cellular concentration of zinc (Fig. 6), indicating that AMT affects selectively the distribution of iron. The decrease of iron concentration following AMT exposure alone is more pronounced in neuronal processes, neurite outgrowths and distal ends, than in cell bodies (Fig. 7). In cells exposed to AMT alone only a basal level of iron is detected with none or very few iron-rich structures (Fig. 2 to [Fig pone-0000925-g003] 4 and 7). Similarly, in cells exposed to iron and AMT, the number of iron-rich structures is lower than in cells exposed to iron alone, especially in neurite outgrowths and distal ends (Fig. 2 to [Fig pone-0000925-g003] 4 and 7).

## Discussion

Dopamine is a neurotransmitter member of the catecholamine family. It is well established that the biosynthesis of dopamine takes place in the cytosol from which the neurotransmitter is transported through a specific transport system into presynaptic vesicles. The vesicular storage protects dopamine from degradation by the enzyme monoamine oxidase. In differentiated PC12 cells, dopamine is known to be stored within large dense core vesicles of 100–200 nm in the cytosol and neuronal processes (dendrites and axons), with greatest intensity at varicosities, branch points and distal ends [15], [18]. This is exactly what we observed in this study using epifluorescence microscopy of dopamine distribution in PC12 cells (Fig. 5). These observations provide the first experimental evidence that iron and dopamine accumulate into dopamine vesicles of dopamine producing neurons.

Potassium shows an intracellular distribution proportional to cell volume (Fig. 2 to [Fig pone-0000925-g003] 4) as expected from the known ubiquitous distribution of this element in mammalian cells [19], [20]. Similarly, zinc is ubiquitous within the cell volume, with slightly higher levels in the nucleus (Fig. 2 to [Fig pone-0000925-g003] 4). It is also well known that this essential trace element is involved in numerous protein functions in all cellular compartments, including zinc-finger transcription factors in the nucleus [21]. Zinc does not co-localize with iron in dopamine neurovesicles which suggests a selective interaction between dopamine and iron.

It is interesting to note that AMT, an inhibitor of dopamine synthesis, had no effect on the cellular concentration of zinc (Fig. 6), indicating that AMT affects selectively the distribution of iron. The decrease of iron concentration following AMT exposure alone is more pronounced in neuronal processes, neurite outgrowths and distal ends, than in cell bodies (Fig. 7). Therefore, the inhibition of dopamine synthesis induces a decrease in total iron cellular content, specifically within dopamine vesicles. This result confirms the storage of iron in dopamine vesicles and also suggests a physiological role of dopamine in the control of iron homeostasis in dopaminergic cells.

The observation of a basal level of iron after inhibition of dopamine synthesis (Fig. 2 to [Fig pone-0000925-g003] 4 and 7) indicates that dopamine vesicles are not the only sites for iron storage. The iron storage protein ferritin is synthesized by PC12 cells [22], [23] and ferritin molecules are known to be present in axons of neuronal cells [24]. Redox metals such as iron do not appear free in solution to any extent in healthy living systems because of their highly toxic reactivity. Both systems, ferritin and dopamine, could contribute to iron storage in dopaminergic cells. In addition, it can be speculated that our observation of iron-dopamine structures in neurite outgrowths could also be related to the axonal transport of iron from the cytosol to the synapse [25], [26].

PD results from a shortage of dopamine in the brain induced by the selective death of dopamine producing neurons in the SNpc [27] Dopaminergic neurons die in a slow but progressive manner leading to a depletion of dopamine in the striatum compromising the capacity of the brain to orchestrate voluntary movement. The causes of the selective death of SNpc dopaminergic neurons in PD are still largely unknown. Increasing evidence suggests that abnormal iron handling in the brain may be involved in PD etiology [1]–[4]. PD is both characterized by iron specific accumulation in the SNpc [3]–[7], and by a decrease in TH protein content and TH mRNA in SNpc dopaminergic neurons relative to control subjects [28], [29]. Our results suggest that the elevation of iron concentration in the SNpc, and the concomitant loss of TH hydroxylase activity, may lead to a lack of iron-dopamine binding capability rendering the dopaminergic neurons more prone to iron toxicity. It has been also suggested that mutations in α-synuclein, a protein mutated in some familial forms of PD, could result in a reduced number of vesicles being available for dopamine storage, leading to an accumulation of dopamine in the cytoplasm and increased levels of oxidative stress [30], [31]. In this context, a mechanism involving iron in PD progression could result from the decrease of dopamine neurovesicles and the redistribution of highly oxidant iron-dopamine compounds in dopaminergic neurons. Using synchrotron X-ray chemical nano-imaging it will now be possible to study iron distribution in cellular models of PD dopaminergic neurons, and more generally, to investigate the subcellular distribution of any metal ion involved either in neurodegenerative diseases, or in physiological neuronal functions.

## Materials and Methods

### Synchrotron X-ray fluorescence chemical nano-imaging

Synchrotron undulator radiation is focused efficiently using dynamically bent graded multilayers set in the Kirkpatrick-Baez geometry [32]. While this geometry is classical in X-ray optics [33], it is here extended, in combination with one of the world most brilliant X-ray sources, to an unprecedented spatial resolution and photon density. Both properties are however required for quantitative mapping of trace elements at the sub-cellular level. The spatial extent of the nanoprobe is below 90 nm in both directions. The experiments were conducted on a long, coherent beamline (ID19) and on a shorter beamline using the concept of a secondary source (the nano-imaging facility ID22NI). The first mirror of the X-ray optical device, coated with a graded multilayer, plays both the role of vertical focusing device and monochromator, resulting in a very high and unique X-ray flux (a few 10^12^ photons/s) at energies between 15 and 17 keV. The sample, mounted in air on a piezo nano-positioner stage, is scanned through the focal plane while the spectrum of the emitted fluorescence is recorded with an energy dispersive Si(Li) detector. The integration time per scan point was varied in the range 300 ms–1 s and kept below the onset of structural radiation damage as verified by repetitive sampling of the same specimen region.

### Quantitative mapping of iron distribution

The X-ray fluorescence recorded spectra are fitted to obtain quantitative maps of the elements expressed in nanograms per square centimeters [34]. The analysis of a certified reference material (Micromatter 4016), consisting in a Zn thin film deposit of 63.3 µg/cm^2^±5%, enabled to calibrate the element concentrations. Iron concentrations in subcellular compartments (cell body, neurite outgrowths, and distal ends) were determined using PyMCA software [35] which enables to extract data from selected zones of the scanned area, and to fit corresponding X-ray fluorescence spectra. Mean iron quantitative distributions and standard deviation of the mean in subcellular compartments were calculated for each culture condition.

### Cell cultures and sample preparation

Rat pheochromocytoma PC12 cells were used as *in vitro* model of dopamine producing cells [14], [15]. PC12 cells were routinely maintained in RPMI 1640 medium, 4.5 g/L glucose, 10% equine serum, 5% fetal bovine serum, 2 mM glutamine and 100 U/mL penicillin-streptomycin, at 37°C in a water-saturated atmosphere containing 5% CO_2_. All chemical and biochemical compounds were from Sigma. About 2.10^4^ PC12 cells were split directly onto 2 cm diameter sample holders consisting in a 2 µm thin polycarbonate foils for synchrotron X-ray fluorescence nano-imaging, or micro-PIXE (particle induced X-ray emission) analysis. Cells were allowed to differentiate with 100 ng/mL nerve growth factor and exposed to 300 µM FeSO_4_ during 24 h, to 1 mM AMT during 96 h, or to both compounds. These AMT and iron concentrations were found sub-cytotoxic as verified on separate experiments by cell counting assays. After appropriate exposure times, PC12 cells were rinsed with phosphate buffer solution, cryofixed at −160°C by plunge freezing into isopentane chilled with liquid nitrogen, and freeze dried at −35°C. This protocol was applied to maintain cellular morphology and chemical element distribution integrity [20].

### Particle induced X-ray emission (PIXE)

Micro-PIXE analysis was performed to obtain quantitative element concentrations on groups of several hundred cells on the same samples to complete synchrotron X-ray fluorescence nano-imaging of single cells. PIXE and Rutherford Backscattering Spectrometry (RBS) were performed simultaneously using the nuclear microprobe beamline at the Centre d'Etudes Nucléaires de Bordeaux Gradignan (CENBG), France. The nuclear microprobe enables quantitative chemical analysis of trace elements in cells [13]. In brief, the energy of the incident proton beam produced by the Van de Graaff accelerator was 2.5 MeV. The beam was focused onto the sample surface to a spot of 5 µm in diameter, resulting in a proton beam current of 250 pA as measured with a Faraday cup below the sample. X-ray fluorescence measurements were made with a Si(Li) energy dispersive detector placed at 45° from the incident beam direction. The RBS measurements were performed using a PIPS detector (passivated implanted planar silicon) placed at 135°. X-ray fluorescence data were analyzed with the Gupix software, used to compute X-ray attenuation within sample and variation in ionization and emission cross sections during slowing down of incoming particles. RBS data were analyzed with SIMNRA code and were used for mass normalization of X-ray emission leading to quantitative results expressed in terms of µg of element per g of sample.

### Dopamine fluorescence microscopy

Dopamine fluorescence was observed immediately after freeze drying using an epifluorescence microscope (Olympus) with a combination of filters (U-MWU2) that coordinates excitation at 320–370 nm and allows observation of emission in the 420–600 nm range. Dopamine fluorescence was observed only in the case of cells exposed to an excess of iron. The observed fluorescence of dopamine is due to the formation of a fluorochrome compound after ring closure of dopamine. Oxidized catecholamines are fluorochromes with reported peak absorption and emission wavelengths respectively at 340–360 nm and 410–440 nm [16]. In the case of dopamine, oxidized dopamine absorbs at 335 nm and emits fluorescence with a maximum at 470 nm [17] which corresponds to the blue fluorescence observed in this study.
